# Supplementary material for: Development of Improved High-Performance Liquid Chromatography Method for the Determination of Residual Caprylic Acid in Formulations of Human Immunoglobulins
Source: Molecules. 2022 Mar 3;27(5):1665. doi: 10.3390/molecules27051665 (PMC8912018; doi:10.3390/molecules27051665)
Supplement: Supplementary file 1 [file molecules-27-01665-s001.zip › molecules-1614287-supplementary.pdf]

Supplementary Materials

Development of improved high-performance liquid chromatography method for the determination of residual caprylic acid in formulations of human immunoglobulins

Adela Štimac [1,*], Tihana Kurtović [1], Nediljko Pavlović [2] and Beata Halassy [1,*]

[1] University of Zagreb, Centre for Research and Knowledge Transfer in Biotechnology, Rockefellerova 10, 10000 Zagreb, Croatia; adela.stimac@unizg.hr
[2] Institute of Immunology, Inc., Rockefellerova 10, 10000 Zagreb, Croatia

*Correspondence: adela.stimac@unizg.hr (A.Š.); bhalassy@unizg.hr (B.H.)

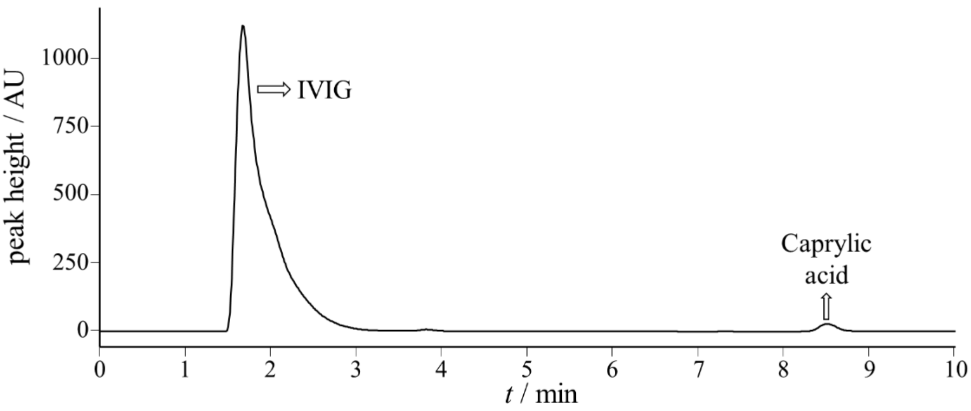

Figure S1. Representative HPLC chromatogram of IVIG sample (1 mg/mL) spiked with caprylic acid (0.5%, v/v). Chromatographic conditions are given in Table 1.

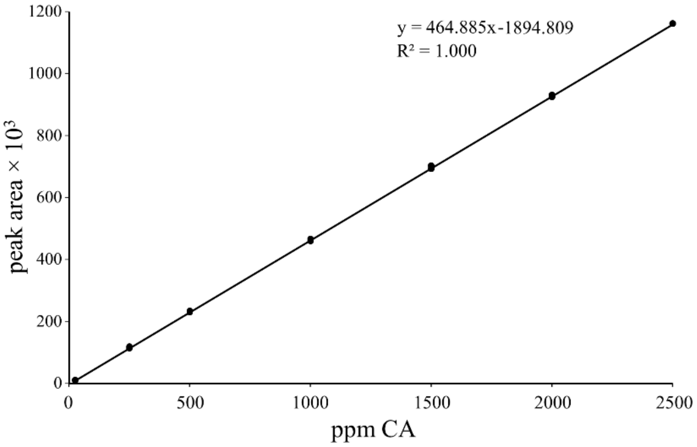

Figure S2. Example of calibration curve of CA.
